# Diagnostic Performance and Geographic Variation in Mammography Services: A Nine-Year Audit from Southern Jordan

**DOI:** 10.3390/diagnostics16162668

**Published:** 2026-08-21

**Authors:** Sanaa Hussein Alnaimat, Norhashimah Mohd Norsuddin, Iza Nurzawani Che Isa, Marwan Alshipli, Ahmad A. Abushattal, Deshinta Arrova Dewi, Shereen A. Alrawadeih, Fahem Kreshan

**Affiliations:** 1 Centre of Diagnostic, Therapeutic and Investigative Studies (CODTIS), Faculty of Health Sciences, Universiti Kebangsaan Malaysia, Kuala Lumpur 56000, Malaysia; zawaniisa@ukm.edu.my; 2 Department of Chemistry, Faculty of Sciences, Al-Hussein Bin Talal University, P.O. Box 20, Ma’an 71111, Jordan; 3 Faculty of Applied Medical Sciences, Al Al-Bayt University, P.O. Box 130040, Mafraq 25113, Jordan; malshipli@aabu.edu.jo; 4 Department of Physics, Al-Hussein Bin Talal University, P.O. Box 20, Ma’an 71111, Jordan; ahmad.abushattal@ahu.edu.jo; 5 Faculty of Data Science and Information Technology, INTI International University, Nilai 71800, Malaysia; deshinta.ad@newinti.edu.my; 6 Department of Medical Imaging, Ma’an Hospital, Jordanian Ministry of Health, Ma’an 71111, Jordan; shereen_rwd@yahoo.com (S.A.A.); aasphy2009@yahoo.com (F.K.)

**Keywords:** mammography, diagnostic performance, breast cancer screening, BI-RADS^®^, public health, southern Jordan

## Abstract

**Background:** Breast cancer continues to be a major contributor to illness among women in Jordan, particularly in underserved regions. Despite the establishment of mammography services in southern Jordan, no formal quality assurance audit has evaluated diagnostic performance or geographic equity of access. This study aimed to evaluate the diagnostic performance of mammography services using established quality assurance indicators and to investigate geographic variation in mammographic findings and diagnostic pathways among women attending a regional referral center in southern Jordan. **Methods:** This study employed a retrospective hospital-based clinical audit that was conducted using mammography records from Ma’an Hospital between 2016 and 2024. A total of 592 women aged 20 years and older who underwent mammography were included. Data extracted from electronic and paper-based medical records included demographic characteristics, mammography indication, BI-RADS^®^ classifications, follow-up procedures, reporting timelines, and histopathological outcomes. Histopathological confirmation served as the reference standard for diagnostic accuracy analyses. Diagnostic performance was evaluated using Cancer Detection Rate (CDR), sensitivity, specificity, positive predictive value (PPV), and negative predictive value (NPV). Geographic variation was evaluated according to place of residence, while multivariable logistic regression was used to determine whether associations remained independent of age and mammography indication. **Results:** Of the 592 mammography examinations, 354 (59.8%) were performed for screening purposes and 238 (40.2%) for diagnostic evaluation. BI-RADS^®^ 1 and BI-RADS^®^ 2 were the most frequently assigned categories, accounting for 38.3% and 28.7% of examinations, respectively, whereas BI-RADS^®^ 4 and 5 findings accounted for 6.3% and 1.0%. The overall CDR was 52.4 per 1000 examinations with sensitivity (93.9% (95% CI 80.4–98.3%)) and specificity (97.4% (95% CI 95.3–98.5%)), positive predictive value (PPV) (73.8% (95% CI 58.0–86.1%)), and negative predictive value (NPV) (99.5% (95% CI 98.2–99.9%)). Suspicious findings (BI-RADS^®^ 4–5) were significantly more frequent among urban than rural women (9.4% vs. 3.7%; adjusted OR 3.05, 95% CI 1.37–6.78), whereas reporting delay, ultrasound use, biopsy rate, and further follow-up did not differ significantly by residence. **Conclusions:** Mammography services at Ma’an Hospital exhibit high diagnostic accuracy and strong clinical performance in breast cancer detection over the nine-year study period. Women in rural areas were less likely to have suspicious mammographic findings detected than those in urban areas, regardless of age and mammography type. No significant differences were observed in reporting timelines or follow-up procedures by place of residence. These findings underscore the value of regional quality assurance audits in identifying service disparities and guiding improvements in breast cancer screening and diagnostic services, especially in underserved communities.

## 1. Introduction

Breast cancer represents the most common type of cancer diagnosed in women across the world, and a considerable public health problem in Jordan. It accounted for approximately 38.5% of total female cancers reported by the Jordan Ministry of Health in 2022 [1,2,3,4,5]. In an effort to increase the rate of early detection of breast cancer, the Jordan Breast Cancer Program (JBCP), which began operations in 2007, has been designed to increase awareness and provide greater access to high-quality screening services. The JBCP guidelines recommend that women begin performing monthly breast self-exams at age twenty, have annual clinical breast examinations between the ages of twenty and thirty-nine, and undergo annual mammograms at age forty [6,7]. However, despite these recommendations, many women are reluctant to seek out breast cancer screenings. Women’s participation in breast cancer screening may be hindered by factors such as financial constraints, cultural misconceptions about screening, and low levels of breast health awareness [8,9]. Although the JBCP offers free mammography vouchers and home-based educational sessions, uptake has not improved substantially [10,11,12,13].

In low- and middle-income countries (LMICs), breast cancer screening is commonly delivered through either organized or opportunistic approaches. Organized screening programs target a defined population, systematically invite eligible women for screening, provide follow-up for abnormal findings, and incorporate quality assurance measures, participant registries, and continuous performance monitoring [14,15,16,17]. In contrast, opportunistic screening occurs when women seek screening independently, are referred by healthcare providers, or participate in awareness and screening campaigns. This is because opportunistic screening, particularly in hospital settings, often includes symptomatic women or those at higher risk. It generally reports higher cancer detection rates than population-based organized screening programs [18,19,20]. Thus, evaluating the mammography services offered at the Ma’an Hospital provides valuable data regarding both the effectiveness and feasibility of providing mammograms within a mixed opportunistic and diagnostic setting in an underserved low- to middle-income country. Although Jordan’s age-standardized cancer incidence rate declined from 59.5 to 34.1 per 100,000 population between 2015 and 2020, breast cancer remains a major public health concern, accounting for 19.7% of cancer-related deaths. Survival rates vary considerably by stage at diagnosis, ranging from 96% for stage I disease to 31.5% for stage IV disease [20,21]. Previous studies in Jordan have primarily focused on prevalence, risk factors, and barriers to screening, with most research conducted in the northern and central regions [17,20]. In contrast, the southern region, particularly Ma’an Governorate, remains understudied and underserved, with limited access to oncology services. Despite higher mortality rates and demographic characteristics compared with other regions, breast cancer screening participation among women in southern Jordan appears to be lower. Women from southern Jordan often experience longer delays in breast cancer diagnosis and treatment than those in other regions. The most recent report from the Ministry of Health indicated lower mammography utilization in southern governorates compared with northern governorates, highlighting persistent geographic disparities in access to breast cancer screening and care. Given its large geographical area and dispersed population, Ma’an provides an important setting for evaluating the quality, accessibility and equity of breast imaging services in an underserved region [22,23,24,25].

Although a dedicated mammography unit was established at Ma’an Government Hospital in 2016, there have been no prior studies conducted on the diagnostic capability, quality assurance criteria, or geographic distribution of access to breast imaging services using internationally recognized standards for breast imaging performance metrics. As such, it remains unclear as to whether women living in this geographic region were receiving breast imaging services meeting accepted quality standards, and/or whether geographic barriers impacted their ability to obtain diagnostic services and receive appropriate follow-up care [26,27,28].

The primary rationale for conducting quality assurance evaluations (QA) of breast imaging services, especially in resource-limited settings and remote/rural areas due to geographic barriers, is to ascertain if service availability translates into patients’ utilization of those services and subsequent timely diagnosis. However, utilization alone is not a sufficient indicator of program success. The screening service must also meet accepted quality standards to achieve its ultimate goals of reducing breast cancer mortality while minimizing harms, including false results, overdiagnosis, and unnecessary radiation exposure. Therefore, regional QA seeks to establish not only whether available services are used, but whether they meet recognized performance benchmarks. Evaluating QA on a regional basis will provide information regarding rates of participation in screening programs, follow-up of abnormalities detected during screening, diagnostic accuracy, and continuity of care. Evaluations of this type can aid in identifying potential service delivery gaps contributing to disparities in breast cancer detection and management among rural residents. Internationally, performance has historically been evaluated through the use of established indicators such as cancer detection rate (CDR), sensitivity and specificity—all based upon BI-RADS@ (Breast Imaging Reporting and Data System) guidelines [29,30,31,32]—which allows for comparisons against well-established standards. Furthermore, the use of these guidelines enables the identification of service delivery areas requiring enhancement. Additionally, in countries where significant inequities exist related to the distribution of healthcare resources, systematic audits are critical for providing the required evidence-based support for quality improvement initiatives and developing national cancer control strategic plans.

## 2. Methodology

### 2.1. Ethical Approval and Study Setting

The study was approved by the Research and Ethics Committee of both the Jordanian Ministry of Health and Universiti Kebangsaan Malaysia (UKM) (study reference number: JEP-2024-979). Data were collected at Ma’an Governmental Hospital, a hospital that acts as a principal referral hospital for all breast imaging in Ma’an Governorate, Jordan. All data collected were de-identified to preserve participants’ privacy prior to analysis.

### 2.2. Study Design and Patient Selection

This study is a retrospective clinical audit that combines diagnostic accuracy and assessment of health services. It used all mammography data collected at Ma’an Governmental Hospital from January 2016 through June 2024. The hospital is the primary source for breast imaging in the Ma’an Governorate; therefore, it offers both screening and diagnostic mammography. The analysis was based solely on the medical record for each woman who underwent an examination at the hospital’s radiology department as part of her routine care. All women eligible must be twenty years or older and live in the Ma’an governorate. They must have either been screened for mammograms or diagnosed with breast cancer via mammogram between study dates. An upper age limit was selected to best represent the demographics of Ma’an, which has large numbers of women who are twenty and above living there [32]. Women whose medical records were incomplete regarding the following—age, medical history or screening results—were eliminated from consideration. Duplicate entries were also deleted to ensure the reliability of the data set. Ultimately, this process resulted in 592 women being evaluated in the context of this audit. The audit utilized the same fundamental principles outlined in STARD for reporting studies assessing diagnostic performance. Diagnostic performance was measured using mammographic assessments utilizing BI-RADS criteria classifications and verified against histopathological confirmation where a tissue diagnosis was available, and against documented clinical follow-up otherwise. As the cohort included both screening and diagnostic (symptomatic) examinations, the cancer detection rate was expected to be higher than that of organized asymptomatic screening programs. Therefore, the diagnostic performance metrics should be interpreted in the context of this mixed cohort.

### 2.3. Classification of Mammography Type

Mammography was divided in this research study into two types based on clinical intention: screening and diagnostic. The primary purpose for conducting screening mammograms is to obtain images of asymptomatic women that may indicate early detection of breast cancer when it can be treated more easily. Conversely, the goal of performing diagnostic mammograms is to produce images that will help diagnose the cause of specific clinical findings such as a palpable mass, nipple discharge, localized tenderness, or a prior abnormality detected during a screening examination.

### 2.4. Data Collection

Mammography data were drawn from the national electronic health record system (Hakeem), which indexes patient medical information by national identification number. The cohort comprised women who underwent mammography at Ma’an Governmental Hospital, whether referred by a physician or presenting on their own for screening. Hard copies from institutions were cross-checked with digital entries for completeness. Hard copy documents were obtained when digital information was missing. Although reconciling paper and electronic records reduced missingness, some records remained incomplete and, as detailed below, were excluded from the final analysis. The socio-demographics included in this study include age, where the woman resided at the time of examination, and her marital status. Procedural data are also included in this study, such as the date of examination, type of mammographic view used, BI-RADS^®^ category assigned by the radiologist for each case examined, and whether she had been recalled or if she is still on active follow-up. All digital entries were carefully checked against corresponding paper records, and any records missing vital information such as patient age, residency, number of mammogram images, or the identity of the interpreting radiologist were removed from the final analysis.

### 2.5. Performance Metrics

Performance indicators were organized into three domains: (1) Diagnostic Performance, encompassing Cancer Detection Rate (CDR), sensitivity, specificity, positive predictive value (PPV), and negative predictive value (NPV); (2) Mammographic Findings, based on BI-RADS^®^ categories; and (3) Access Indicators, including reporting delay and place of residence (urban vs. rural).

**Cancer Detection Rate** (CDR) represents the number of breast cancer cases identified among the screened population. This metric quantifies the number of malignant cases identified per 1000 examinations. It is calculated as:

**CDR = (True Positives (TP)/Total Number of Screened Women) × 1000**

**Sensitivity** represents the proportion of cancer cases correctly identified by mammography. It is defined as:

**Sensitivity = TP/(TP + FN) × 100**

**Specificity** represents the proportion of non-cancer cases correctly identified by mammography. It is defined as:

**Specificity = TN/(FP + TN) × 100**

**Positive Predictive Value (PPV)** represents the proportion of mammograms classified as positive (BI-RADS^®^ 4 or 5) that were confirmed malignant on histopathology. It is defined as:

**PPV = TP/(TP + FP) × 100**

**Negative Predictive Value (NPV)** represents the proportion of mammograms classified as negative (BI-RADS^®^ 1 or 2) in women subsequently confirmed to be free of malignancy. It is defined as:

**NPV = TN/(TN + FN) × 100**

**Mammographic Findings.** Radiological findings were summarized according to BI-RADS^®^ category (0–5), as assigned by the reporting radiologist. The distribution of BI-RADS^®^ categories and their association with follow-up procedures were examined to evaluate recall patterns and clinical decision-making.

**Access Indicators.** Geographic disparities in access were assessed using two indicators. **Reporting delay** was defined as the number of days between the mammography examination date and the date on which results were communicated to the patient, with delays exceeding seven days considered clinically significant. **Residence** was classified as urban or rural based on patient address recorded at the time of examination and was used to stratify all access-related analyses.

Classification was based on histopathological confirmation as the gold standard. A True Positive (TP) was defined as a BI-RADS 4 or 5 finding confirmed as malignant, while a False Positive (FP) occurred when these categories yielded benign histopathology. A True Negative (TN) represented BI-RADS 1 or 2 findings in patients who remained cancer-free, whereas a False Negative (FN) described cases initially categorized as BI-RADS 1, 2, or 3 that were later confirmed as malignant. As Ma’an Governmental Hospital provides breast imaging services but does not offer on-site surgical oncology or definitive histopathology, women with suspicious findings (BI-RADS 4 or 5) were referred to tertiary centers for image-guided core-needle biopsy or surgical excision. The follow-up procedure recorded reflects only the step initiated at Ma’an at the index visit and therefore under-represents the biopsies ultimately completed after referral, which are captured in the national electronic health record (Hakeem) under the receiving center rather than as a Ma’an procedure. A finding was classified as a true positive only when a BI-RADS 4 or 5 examination was matched to a definitive malignant histopathology report in Hakeem, irrespective of where the tissue was obtained, and as a false positive when such an examination yielded benign histopathology. A woman was counted as a true negative only when a BI-RADS 1 or 2 index examination was accompanied by at least 12 months of subsequent documented follow-up in Hakeem with no recorded breast-cancer diagnosis; BI-RADS 1 or 2 examinations with insufficient follow-up to satisfy this window were treated as indeterminate and excluded from the accuracy denominator. As the service does not operate a mandated recall system or cancer-registry linkage, interval cancers among women who did not re-present cannot be fully excluded, and the reported sensitivity and negative predictive value should accordingly be interpreted as upper-bound estimates.

### 2.6. Radiological Classification

Radiological findings were evaluated based on BI-RADS^®^ classification. To enable clinical decision-making and follow-up, the reporting radiologist assigns BI-RADS^®^ categories to each mammogram (range 0 to 5). See Table 1: the BI-RADS^®^ Classification of Mammography Findings [1].

### 2.7. Data Analysis

Statistical analysis was performed using IBM SPSS Statistics version 26 supported by Microsoft Excel. Descriptive statistics were employed to summarize patient demographics, including age and place of residence, as well as clinical variables. Diagnostic performance indicators (CDR, sensitivity, specificity, PPV, NPV) were calculated with 95% confidence intervals using the Clopper–Pearson exact method for sensitivity and specificity, and the Poisson exact method for CDR. The normality of continuous data was assessed using the Shapiro–Wilk test. Categorical variables were compared using Chi-Square or Fisher’s Exact tests as appropriate. Reporting delays were compared between urban and rural residents using the Mann–Whitney U test. Annual trends in diagnostic performance were examined descriptively across the study period.

## 3. Results

### 3.1. Participant Characteristics

A total of 592 mammography examinations performed between January 2016 and June 2024 met the study inclusion criteria. The mean age of participants was 47.8 ± 8.6 years (range: 27–95 years), with the highest proportion of women belonging to the 45–49-year age group. Urban residents accounted for 373 examinations (63.0%), whereas 219 examinations (37.0%) were performed in women residing in rural areas. Regarding clinical indication, 354 examinations (59.8%) were classified as screening mammography and 238 examinations (40.2%) as diagnostic mammography. Table 2 summarizes the demographic and clinical characteristics of the study cohort.

### 3.2. Mammography Findings and Follow-Up Procedures

The mammographic findings of the 592 examinations were classified according to the Breast Imaging Reporting and Data System (BI-RADS^®^). BI-RADS^®^ 1 (negative findings) was the most frequently assigned category, accounting for 227 examinations (38.3%); however, a substantial proportion of examinations (21.6%) were classified as BI-RADS^®^ 0, indicating incomplete assessment requiring additional imaging evaluation. BI-RADS^®^ 2 (benign findings) was assigned in 170 examinations (28.7%). BI-RADS^®^ 3 (probably benign) was assigned to 24 examinations (4.1%), while BI-RADS^®^ 4 (suspicious abnormality) and BI-RADS^®^ 5 (highly suggestive of malignancy) were reported in 37 (6.3%) and 6 (1.0%) examinations, respectively (Table 3).

Follow-up diagnostic procedures were performed according to the radiological findings. Ultrasound was the most frequently utilized additional imaging modality, performed in 288 cases (48.7%). Routine follow-up mammography was recorded in 134 cases (22.6%), while no additional diagnostic procedure was required in 152 cases (25.7%). Biopsy procedures were performed in 15 cases (2.5%), and repeat mammography was performed in 3 cases (0.5%) (Table 4).

The relationship between BI-RADS^®^ classification and follow-up procedure was further evaluated. A statistically significant association was observed between the two variables (χ^2^ = 305.24, df = 20, *p* < 0.001), indicating that the type of follow-up investigation varied according to BI-RADS^®^ category. Higher BI-RADS^®^ classifications were associated with more intensive diagnostic assessment, including ultrasound examination and tissue biopsy (Table 5). Among the 24 women assigned BI-RADS 3, 19 (79.2%) returned for the recommended 6-month short-interval reassessment by follow-up ultrasound, while the remaining 5 (20.8%) had no documented follow-up in Hakeem and were treated as censored and excluded from the false-negative analysis; none of the BI-RADS 3 women with recorded follow-up progressed to malignancy within the audit window. Table 5 additionally shows that the large volume of supplementary imaging is driven predominantly by BI-RADS 0 examinations (97 ultrasounds) and, to a lesser extent, by BI-RADS 4 and 5 findings; the aggregate follow-up counts in Table 4 should therefore be read together with this category-specific breakdown.

### 3.3. Diagnostic Performance

**Figure 1:**
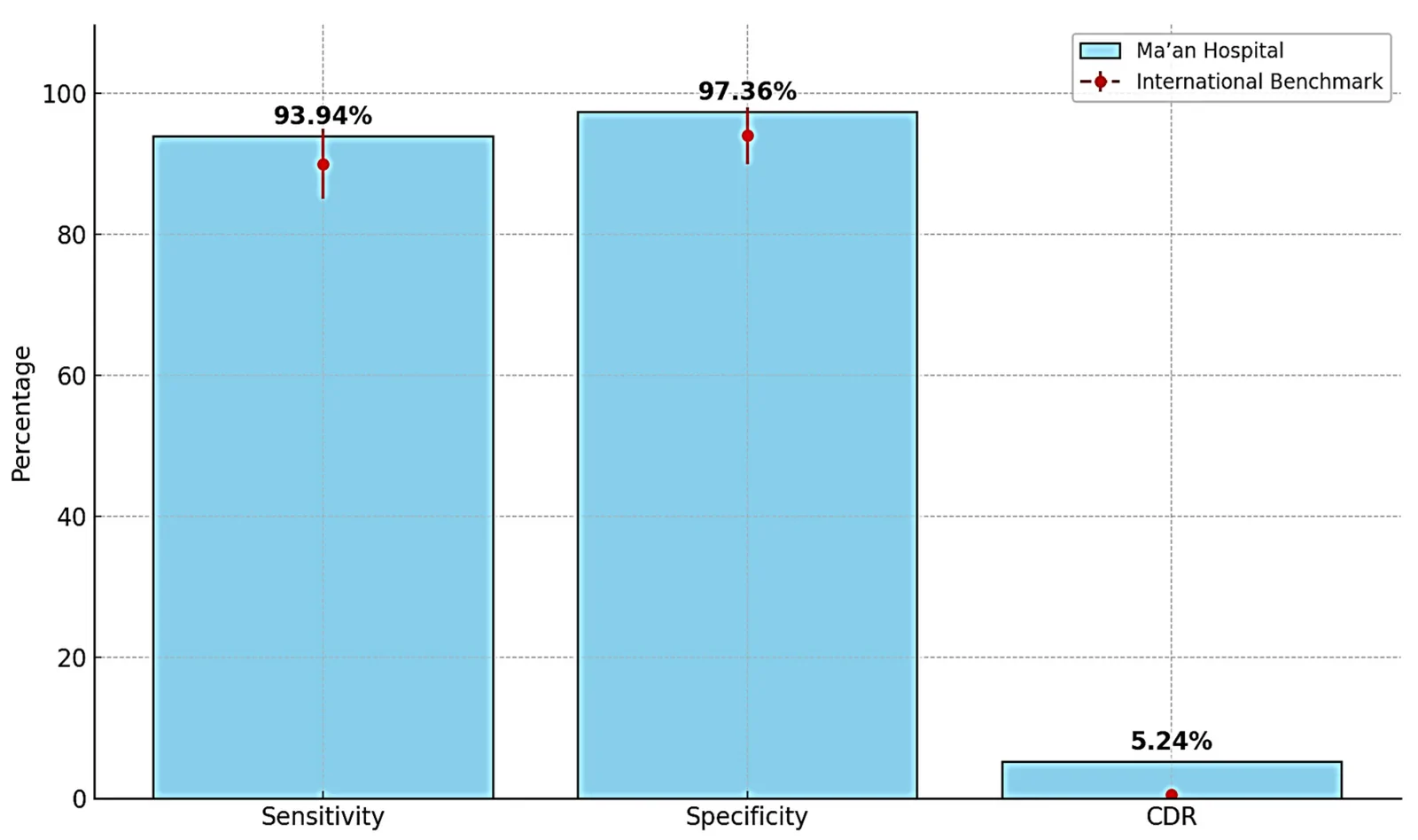
The comparison of diagnostic metrics, Ma’an Hospital versus international benchmarks (Benchmark Ranges for Breast Screening Programs in Norway and Vermont, USA [33] and the United States and Denmark) [34]).

Of the 592 mammography exams that were part of this study, 33 patients had confirmed breast cancer by histopathology and served as the reference standard for the subset of women in whom a tissue diagnosis was available, whether obtained on-site or after referral (Section 2.5). For the remaining women, final classification relied on documented clinical follow-up. Mammography resulted in correct identification of 31 malignant cases as true positives, and incorrectly identified two malignant tumors (BI-RADS 1 or 2 at time of exam), which were later confirmed via follow-up as false negatives. In addition to the two false negatives, there were 11 false positives among the total of 452 remaining exams, and 406 were true negatives. This produced an overall cancer detection rate (CDR) of 52.36/1000. The sensitivity of mammography was found to be 93.94%, indicating that most breast cancers were detected; specificity was 97.36%, showing that mammography is able to rule out women who do not have malignancy very well. Positive predictive value was 73.81% (95% CI = 58.0–86.1%), and the negative predictive value was 99.51% (95% CI = 98.2–99.9%) see Figure 1. Diagnostic performance indicators are summarized in Table 6. Exams designated as BI-RADS 0 (i.e., insufficient for final assessment based upon need for additional imaging), or those lacking adequate follow-up data to determine a final diagnosis, were removed from consideration when determining the sensitivities, specificities, and predictive values. Thus, all three of these measures were calculated using data from the final outcomes for the 450 exams that did not include missing information (31 true positives, 2 false negatives, 11 false positives, and 406 true negatives).

### 3.4. Annual Trends in Diagnostic Performance

Diagnostic Performance Indicators for Mammography Unit from 2016 through 2024 were reported in Table 7. The number of breast exams increased dramatically during the time frame of this study. From an initial eight in 2016, by 2024, there were 112 breast exams performed each year (*p* < 0.001, Spearman ρ = 0.97). For all but two years (in 2019 and 2020, when the sensitivity rate fell to 75.0% due to false negatives on three separate instances), the sensitivity rates remained at 100%. The non-statistical difference found in the Kruskal–Wallis H test indicated that although there may have been slight variations from one year to another, there was no statistical significance (H = 6.45, *p* = 0.597). As for specificity, it remained uniformly high, ranging from 96.8% to 100.0%, again showing no statistical change (H = 1.38, *p* = 0.994). Positive predictive values fluctuated between 60.0% and 100.0% (Spearman ρ = −0.64, *p* = 0.063); Negative Predictive Values remained higher than 98.0% for each year. The range for cancer detection ratios (CDRs) was 44.64 to 125.00 per 1000 screenings and exhibited a negative correlation (Spearman ρ = −0.77, *p* = 0.016), which is indicative of program maturation and also increasing numbers of screening examinations being taken from a less risky population. Overall diagnostic performance continued to be strong throughout the duration of this study.

### 3.5. Comparison of Imaging Findings by Mammography Indication

Of the 592 examinations in the study, 354 (59.8%) were performed for screening and 238 (40.2%) for diagnostic evaluation of symptomatic women. BI-RADS^®^ classifications were distributed differently between these two indications, a difference that reached statistical significance (χ^2^ = 12.99, df = 5, *p* = 0.024). Negative findings (BI-RADS^®^ 1) were more frequently observed among screening examinations (43.2% vs. 31.1%), whereas BI-RADS^®^ 4 (suspicious abnormality) was proportionally more common among diagnostic examinations (7.1% vs. 5.6%). Diagnostic mammography also demonstrated a higher frequency of BI-RADS^®^ 0 (26.1% vs. 18.6%) and BI-RADS^®^ 3 (5.9% vs. 2.8%) findings, consistent with the more heterogeneous clinical presentations triggering diagnostic referral. The distribution of BI-RADS^®^ classifications according to mammography indication is summarized in Table 8.

Diagnostic outcomes were further compared by mammography indication (Table 9). BI-RADS^®^ 4–5 findings occurred at a similar rate in both groups (6.8% screening vs. 8.0% diagnostic, *p* = 0.629). Biopsy, however, was performed far more often in the diagnostic group than in the screening group (5.0% vs. 0.8%, *p* = 0.002), which fits with diagnostic mammography being requested mainly for women who already had a clinical concern. Ultrasound use was broadly similar between the two groups (52.1% vs. 46.3%, *p* = 0.180)

### 3.6. Geographic Variation in Mammographic Findings and Diagnostic Pathways

**Figure 2:**
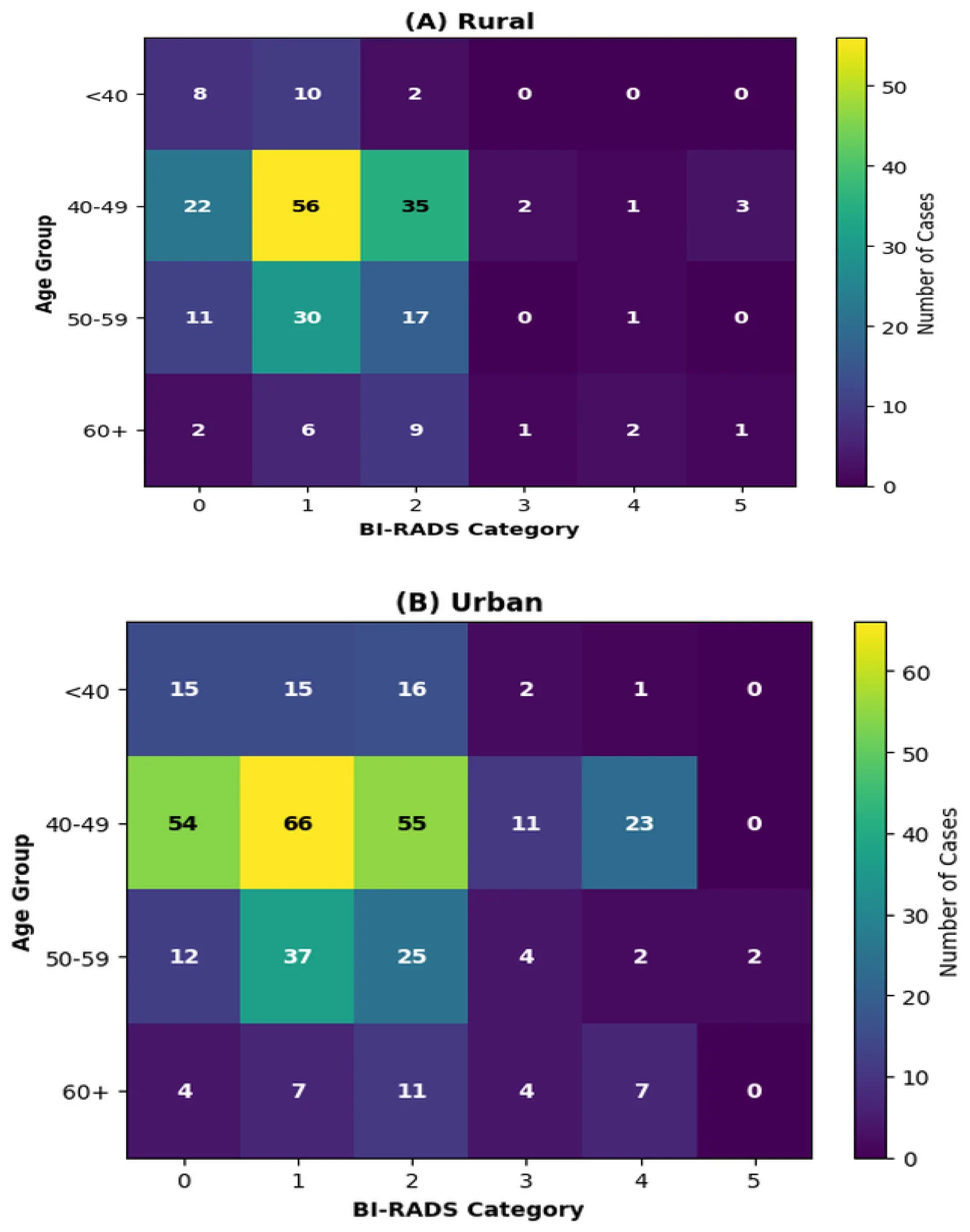
Distribution of BI–RADS classifications across age groups according to residence. (**A**) Rural residents. (**B**) Urban residents. Color intensity represents the number of cases.

Among the 592 women included in the study, 373 (63.0%) resided in urban areas, and 219 (37.0%) resided in rural areas. The difference in location among individuals who were enrolled in this project may be due to variations by population density; access to health care; how referrals are made; use of mammograms; etc. In addition, although there was availability of mammography at Ma’an Governmental Hospital during the entire duration of the project, rural women were significantly underrepresented compared to urban residents. BI-RADS^®^ classifications also varied by place of residence, and heatmap analyses showed how mammographic findings shifted across age groups and residential settings (Figure 2). Suspicious findings (BI-RADS^®^ 4–5) were more frequent among urban than rural women, with urban residence carrying higher odds of a suspicious result (OR = 2.91, 95% CI: 1.32–6.42, *p* = 0.010). In both groups, however, negative and benign findings (BI-RADS^®^ 1–2) remained the dominant categories. However, the relative distribution of higher-risk categories varied according to residential location, indicating differences in the mammographic profiles of women attending the service.

### 3.7. Reporting and Follow–Up Timelines

Most mammography examinations were reported within seven days of image acquisition. Reporting delays exceeding seven days were observed in 49.3% of urban women and 42.9% of rural women, a difference that was not statistically significant (*p* = 0.155). Differences were observed in the distribution of BI-RADS^®^ classifications according to residence. Contrary to the pattern often reported in underserved settings, suspicious findings (BI-RADS^®^ 4–5) were more frequent among urban residents (9.4%) than among rural residents (3.7%) (Table 10), and urban women had significantly higher odds of a suspicious finding than rural women (OR = 2.91, 95% CI: 1.32–6.42, χ^2^ = 6.64, *p* = 0.010) (Table 11). This analysis was restricted to the 464 examinations with a completed risk classification (BI-RADS^®^ 1–5: 288 urban, 176 rural). The 128 examinations classified as BI-RADS^®^ 0 (incomplete assessment requiring additional imaging) were excluded from both groups because they cannot be assigned to a risk category, and rural residence was used as the reference category for the odds ratio. No statistically significant association was identified between residence and the type of follow-up diagnostic procedure performed, including ultrasound (*p* = 0.231), biopsy (*p* = 0.278), or no further procedure (*p* = 0.805).

These heatmaps in Figure 2 present the frequency of BI-RADS classifications within each age group and allow visual comparison between rural and urban populations.

### 3.8. Statistical Associations

**Figure 3:**
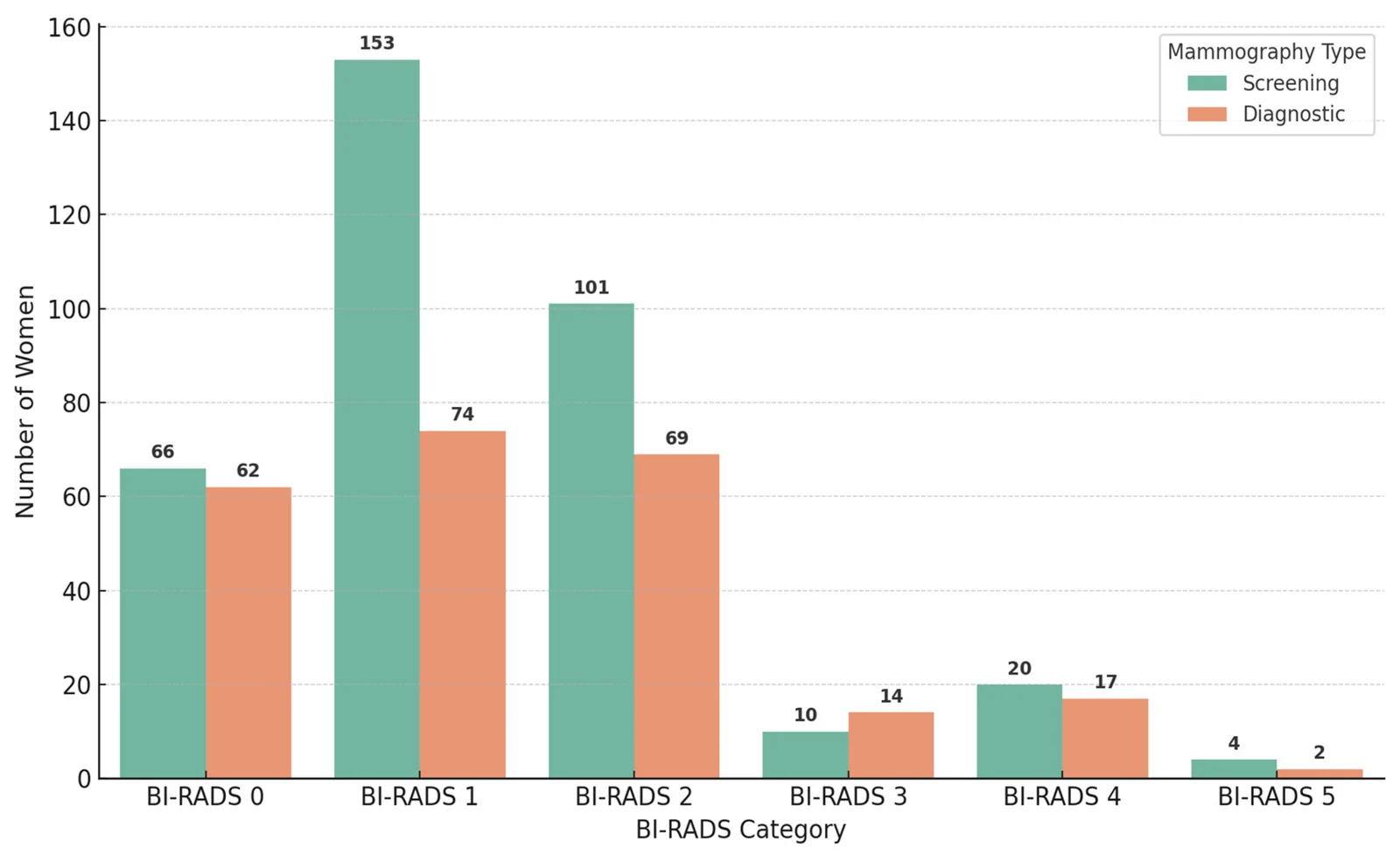
Distribution of BI-RADS Categories by Mammography Type.

To assess whether the association between residence and suspicious mammographic findings (BI-RADS^®^ 4–5) was independent of age and mammography indication, a multivariable logistic regression model was fitted among the 464 examinations with a completed risk classification (BI-RADS^®^ 1–5; BI-RADS^®^ 0 excluded). After adjustment for age and mammography type (screening vs. diagnostic), urban residence remained independently associated with suspicious findings (adjusted OR = 3.05, 95% CI 1.37–6.78, *p* = 0.006), with rural residence as the reference category. Older age was also independently associated with suspicious findings (adjusted OR = 1.04 per year, 95% CI 1.00–1.07, *p* = 0.028). Mammography type was not significantly associated with suspicious findings after adjustment (adjusted OR = 1.30, 95% CI 0.69–2.48, *p* = 0.419). These findings indicate that the urban–rural difference in suspicious BI-RADS^®^ findings observed in the bivariate analysis (Table 12) persisted after accounting for age and clinical indication see Figure 3.

## 4. Discussion

This study represents the first comprehensive evaluation of mammography service performance in southern Jordan. It demonstrates that high diagnostic accuracy can be achieved within a geographically underserved region. The nine-year history of mammography at Ma’an Governmental Hospital has provided a level of experience with similar sensitivity and specificity rates found in established international breast imaging programs. There were statistically significant differences in mammographic results from urban versus rural women; however, there were no statistical differences in the time it took to report their findings and follow up on these patients based upon where they resided. These differences may reflect variations in how likely women are to have a mammogram rather than differences in how these cases are followed up after the mammogram. The sensitivity (93.9%) and specificity (97.4%) in this study indicate high-quality diagnostic performance. The rates of both parameters fall within the ranges reported for most organized breast imaging programs throughout Europe and North America. Sensitivity for such programs ranges from approximately 80% to 95%, and specificity ranges from approximately 90% to 98%. A number of possible factors contributed. Ma’an Governmental Hospital functions as a regional referral hospital; therefore, many mammograms were performed on symptomatic women or those suspected of having a diagnosis of cancer. The likelihood of detecting cancer during a mammogram is increased when the examination is performed on symptomatic women or women who are suspected of having a diagnosis of cancer compared to asymptomatic women undergoing routine screening. Additionally, because histopathologic confirmation was used as the gold standard for determining true positive and false negative outcomes, the degree of verification for each case was maximized, thus minimizing potential errors due to incorrect classification of outcomes [35].

The cancer detection rate of 52.4 cancers detected per 1000 examinations was substantially greater than that reported by population-based screening programs. However, this finding should be viewed as part of the characteristics of the study population. Approximately 40% of these examinations were ordered for diagnostic purposes versus routine screening. Therefore, a substantial number of women underwent mammography due to the presence of symptoms or clinical abnormality. It has been demonstrated repeatedly that mixed screening-diagnostic cohorts produce significantly higher detection rates than do well-organized screening programs solely on the basis that there are more cases of disease present in individuals undergoing examination [36,37]. Consequently, the elevated rate found in this audit would appear to represent both actual diagnostic performance and an increased risk of developing cancer in the cohort referred for evaluation. Importantly, the similar proportion of BI-RADS 4–5 findings between our screening and diagnostic subgroups (Table 9; 6.8% vs. 8.0%, *p* = 0.629) does not conflict with this interpretation. Both subgroups are drawn from a self- and physician-referred hospital population and carry a higher pre-test probability of disease than the asymptomatic, invitation-based cohorts enrolled in organized programs, so the elevated detection rate reflects the character of the whole referred population rather than the diagnostic fraction alone. Comparisons of our cancer detection rate and diagnostic metrics against organized-screening benchmarks should therefore be interpreted with this difference in source population in mind.

Another key quality assurance issue identified through this audit was the fact that 21.6% (more than one out of five) of evaluations required additional imaging before an interpretive assessment could be rendered definitively. This exceeds the rates typically observed in organized screening programs and could result from a variety of factors including dense breast tissue, lack of prior films for comparison, problems with image acquisition, and variation in radiologic interpretation [38,39]. The rate at which BI-RADS 0 assessments occur is important because it contributes to patient anxiety, added cost to the health care system, and delay in obtaining a final diagnosis. A variety of strategies may assist in reducing the rate of BI-RADS 0 assessments while maintaining adequate diagnostic sensitivity, such as monitoring BI-RADS distribution, auditing image quality, implementing peer review processes for radiologists, and providing ongoing continuing education opportunities. In our relatively young cohort (mean age 47.8 years), dense fibroglandular tissue and the absence of prior comparison films in a service established only in 2016 are likely to be the principal contributors, compounded by single-reader interpretation. Because more than one in five examinations received this category, the BI-RADS 0 rate is not only a source of patient anxiety, additional cost and diagnostic delay but also a concrete, actionable target for workflow optimization, achievable through systematic retrieval of prior images, on-table review before patient discharge, selective double reading or arbitration, and continued radiographer training.

The audit also revealed significant geographic variability in the utilization of breast imaging services. While rural women represented only 37.0% of total examinations, they formed a large portion of the hospital’s population and thus may reflect differential access to services or screening rates. Urban and rural residents had comparable reporting times for BI-RADS 2–3 lesions (although this does not preclude there being a reporting delay), but, consistent with the literature, suspicious BI-RADS 4–5 findings were found in higher proportions among urban women [40,41,42]. While it is likely that rural women have an equal or greater likelihood of having breast cancer than their urban counterparts, it is far more plausible that the difference in findings relates to differing usage rates of mammography, differences in referral patterns, health care accessibility and/or participation in preventive screening. Similar geographic differences in mammography usage and screening rates are documented elsewhere in low- and middle-income countries where women residing in distant/remote locations frequently experience obstacles to accessing timely breast imaging/diagnosis—i.e., lack of transportation options to receive breast imaging, limitations of available health resources, and decreased health literacy among others [43,44]. Since the specific causes for the observed urban/rural differences were not assessed directly in this study, additional studies examining healthcare access, screening participation and patient-level socioeconomic determinants would need to be conducted to identify and address inequitable screening practices for breast cancer detection in Southern Jordan. These geographic findings should be interpreted with caution: socioeconomic status, healthcare accessibility, referral pathways, and screening participation were not directly measured in this audit, and the urban–rural contrast cannot be attributed to any underlying difference in disease prevalence. Additionally, these findings support the implementation of focused outreach efforts, the development of mobile mammographic screening programs, and enhanced referral processes to equitably distribute access to breast imaging services throughout both urban and rural populations.

These findings are consistent with prior research from Jordan [20] as well as other middle-income countries [40]; however, most prior research from Jordan [20] has primarily centered on issues related to screening participation awareness/knowledge and barriers to screening participation. Few studies have examined how clinical mammography services function in terms of performance [45,46]. Thus, the present audit provides important practical information regarding service quality.

Similar studies from Malaysia and other low- and middle-income settings have reported that opportunistic and hospital-based screening programs frequently achieve higher cancer detection rates than organized population-based programs because symptomatic women comprise a substantial proportion of participants [41,42]. In such settings, screening programs frequently include symptomatic referrals, which naturally raises the observed detection rate compared with population-based screening programs targeting asymptomatic women. The present findings support these observations and demonstrate that high-quality mammography services can be successfully implemented even in geographically remote settings.

Our findings indicate clear geographic variation in mammographic findings between rural and urban women in southern Jordan, although reporting delay, ultrasound use, biopsy rate, and further follow-up did not differ significantly by residence. Rural women were substantially underrepresented among service users (37.0% of examinations), and suspicious BI-RADS^®^ 4 and 5 findings were detected less frequently among rural than urban women. Rather than indicating a lower disease burden, one possible explanation is that fewer rural women with symptomatic or advanced disease are reaching mammography services. However, the present study did not directly assess stage at diagnosis, healthcare-seeking behavior, or access barriers, and reporting delay and follow-up patterns did not differ significantly by residence, so this interpretation remains speculative. Similar observations have been reported in underserved populations, where limited access to healthcare services, transportation challenges, and reduced participation in preventive screening contribute to later clinical presentation and diagnostic evaluation [43,44]. The lower proportion of BI-RADS 4 and 5 findings among rural women should not be interpreted as evidence of better breast health in this group.

A number of factors indicate that some rural women are delayed in getting diagnosed due to systemic problems in their health system as well as barriers to accessing primary care and a lower rate of participation in routine mammography. Prior research conducted in Jordan indicates that there are several factors related to socioeconomic status, health literacy, fear of being diagnosed with breast cancer, and culture-specific attitudes toward breast exams that can significantly influence a woman’s decision to seek breast cancer screening or delay seeking medical treatment [45,46]. If similar factors are causing the disparity found in this study, then a focused effort should be made to educate rural women about breast health, increase screening rates among them, and provide continuity of care for them through use of technologies such as telemedicine.

The implications of this study for health planners in Jordan are multifaceted. The hospital’s high level of diagnostic performance at Ma’an Governmental Hospital indicates that regionally provided breast imaging services can produce results equivalent to international standards. However, the gap observed among rural women requires an intentional strategy of outreach, including using mobile mammography units, providing patient navigators, and creating clearer pathways for referring patients from one site to another. Additionally, improving digital methods of communication and recall systems will reduce delays associated with follow-up appointments and enhance the continuum of care. All of the above strategies have the potential to lead to early diagnosis and therefore better outcomes from breast cancer for women living in areas where resources are limited.

The study has significant strengths: It was the first formal evaluation of the quality of mammography performed in southern Jordan; it evaluated data collected over a 9-year time frame; and it used confirmed histopathology for cancer diagnoses. A few limitations affect its overall value. Due to the retrospective nature of the study, there was very little control over completeness of data collection and variable selection. Since not each of the women had their cases verified via histopathology, the possibility exists for verification bias. Data related to breast density, socioeconomic status, education, transportation options available to each participant, and interval cancers were unavailable, thereby limiting explanations regarding geographic differences and possibly inflating the estimates of diagnostic performance. Finally, since the study took place at a single location (hospital), generalization to other locations is problematic. Combining screening and diagnostic studies limits the ability to directly compare this study to studies utilizing organized screening protocols. Because histopathological confirmation was available only for women who proceeded to biopsy or surgery, predominantly those with suspicious imaging, the diagnostic-accuracy estimates are subject to verification (work-up) bias, which tends to inflate both sensitivity and specificity. The audit was designed around clinical performance indicators and did not include a formal technical quality-control dataset (phantom image scoring, mean glandular dose, compression force or image-quality audits); the absence of documented equipment-performance and dose data is a further limitation and an important component for future audits to incorporate. Finally, in the absence of a mandated recall system or cancer-registry linkage, interval cancers among women who did not re-present may be under-ascertained, which would further inflate the estimated sensitivity and negative predictive value.

## 5. Conclusions

The nine-year review is the first comprehensive review of the mammography services provided by Ma’an Governmental Hospital in Southern Jordan. The hospital is one of the most important referral centers for the region. In terms of the ability to detect breast cancer, this review found that mammography was highly sensitive and specific (i.e., it correctly identified almost all breast cancers). The review also showed large regional disparities in mammographic findings. For example, rural women were under-represented among those who used the mammography service, and they had fewer mammograms showing suspicious patterns classified as BI-RADS ^®^. Why there are such large disparities in the use of mammography is not known. However, the results do suggest that future research into how mammography is utilized, into screening participation, and into health care access among rural populations may provide answers.

This review should be interpreted within the context of its retrospective design, and the fact that both screening and diagnostic mammographies have been included. It is difficult to make comparisons using such a design with other population-based screening programs. Despite the limitations of this review, it provides some useful evidence regarding the performance of breast imaging from an area that has a lack of information. Future reviews will benefit from multi-center databases, standardized measures for assessing quality assurance, and multivariate analysis to assess how socioeconomic status and geography affect accessibility to screening. Strengthening rural outreach, follow-up systems, and screening participation may help reduce disparities and improve early breast cancer detection across Jordan.

## Figures and Tables

**Table 1:** BI-RADS^®^ Classification of Mammography Findings [1].

Category	Definition	Recommended Action
0	Incomplete assessment	Obtain additional imaging
1	Negative	Continue routine screening
2	Benign findings	Continue routine screening
3	Probably benign	Short-term follow-up (6 months)
4	Suspicious abnormality	Perform biopsy
5	Highly suggestive of malignancy	Take appropriate clinical action

**Table 2:** Demographic and Examination Characteristics of the Study Participants.

Variable	*n* (%)
Total participants	592
Mean age (years)	47.82 ± 8.61
Urban residence	373 (63.0)
Rural residence	219 (37.0)
Screening mammography	354 (59.8)
Diagnostic mammography	238 (40.2)

**Table 3:** BIRADS distribution in mammography findings (*n* = 592).

BI-RADS Category	*n*	%
0	128	21.6
1	227	38.3
2	170	28.7
3	24	4.1
4	37	6.3
5	6	1.0

**Table 4:** Follow-Up Procedures Performed After Mammography (*n* = 592).

Follow-Up Procedures	*n*	%
No further procedure	152	25.7
Ultrasound	288	48.7
Routine follow-up mammography	134	22.6
Biopsy	15	2.5
Repeat mammography	3	0.5

**Table 5:** Association between BI-RADS^®^ classification and follow-up diagnostic procedures.

BI-RADS^®^ Category	No Further Procedure	Ultrasound	Routine Follow-Up Mammography	Biopsy	Repeat Mammography	Total
0	23	97	4	3	1	128
1	62	50	114	0	1	227
2	53	100	16	0	1	170
3	5	17	0	2	0	24
4	4	23	0	10	0	37
5	5	1	0	0	0	6
Total	152	288	134	15	3	592

Chi-square test: χ^2^ = 305.24, df = 20, *p* < 0.001.

**Table 6:** Overall Diagnostic Performance of Mammography Services.

Indicator	Value	95% Confidence Interval (CI)
Total examinations	592	_
Histologically confirmed cancers	33	_
True Positive (TP)	31	_
False Positive (FP)	11	_
True Negative (TN)	406	_
False Negative (FN)	2	_
Cancer Detection Rate (CDR)	52.36 per 1000	_
Sensitivity	93.94%	80.4–98.3%
Specificity	97.36%	95.3–98.5%
Positive Predictive Value (PPV)	73.81%	58.0–86.1%
Negative Predictive Value (NPV)	99.51%	98.2–99.9%

**Table 7:** Annual Diagnostic Performance Indicators of Mammography Services (2016–2024).

Year	Total Screened	True Positives (TP)	False Positive (FP)	True Negative (TN)	False Negatives (FN)	False Negatives (FN)	Sensitivity (%)	Specificity (%)	PPV (%)	NPV (%)	CDR (/1000)
2016	8	1	0	7	0	0	100	100	100	100	125
2017	24	2	0	22	0	0	100	100	100	100	83.33
2018	45	3	1	41	0	0	100	97.6	75	100	66.66
2019	66	3	2	60	1	1	75	96.8	60	98.36	45.45
2020	58	3	1	53	1	1	75	98.2	75	98.15	51.72
2021	84	4	1	79	0	0	100	98.8	80	100	47.61
2022	98	5	2	91	0	0	100	97.9	71.43	100	51.02
2023	97	5	2	90	0	0	100	97.8	71.43	100	51.54
2024	112	5	2	105	0	0	100	98.1	71.43	100	44.64
Total	592	31	11	406	2	2	93.9%	97.4%	73.8%	99.5%	52.4/1000
*p*-value	<0.001	-	-	-	-	-	0.597	0.994	0.063	0.815	0.016

**Table 8:** Distribution of BI-RADS^®^ Classifications According to Mammography Indication.

BI-RADS^®^ Category	Screening	Diagnostic	Total
0	66	62	128
1	153	74	227
2	101	69	170
3	10	14	24
4	20	17	37
5	4	2	6
Total	354	238	592

Chi-square test: χ^2^ = 12.99, df = 5, *p* = 0.024.

**Table 9:** Diagnostic Outcomes According to Mammography Indication.

Variable	Screening *n* (%)	Diagnostic *n* (%)	*p*-Value
BI-RADS 4–5	24 (6.8%)	19 (8.0%)	0.629
Biopsy performed	3 (0.8%)	12 (5.0%)	0.002
Ultrasound performed	164 (46.3%)	124 (52.1%)	0.180

**Table 10:** Comparison of Diagnostic Pathways According to Residence.

Variable	Urban *n* (%)	Rural *n* (%)	*p*-Value
Participants	373 (63.0)	219 (37.0)	-
Delay ≤ 7 days	189 (50.7)	125 (57.1)	-
Delay > 7 days	184 (49.3)	94 (42.9)	0.155
BI-RADS 4–5	35 (9.4)	8 (3.7)	0.015
Ultrasound follow-up	189 (50.7)	99 (45.2)	0.231
Biopsy	12 (3.2)	3 (1.4)	0.278
No additional procedure	94 (25.2)	58 (26.5)	0.805

**Table 11:** Residence versus BI-RADS Risk Category.

Residence	BI-RADS 1–3	BI-RADS 4–5
Urban	253	35
Rural	168	8

Chi-square: χ^2^ = 6.64, *p* = 0.010. BI-RADS^®^ 0 (incomplete assessment; *n* = 128) was excluded from both groups because it cannot be classified as a risk category; rural residence is the reference category for the odds ratio.

**Table 12:** Multivariable Logistic Regression for Suspicious Findings (BI-RADS^®^ 4–5 vs. 1–3), *n* = 463.

Variable	Adjusted OR	95% CI	*p*-Value
Residence (Urban vs. Rural)	3.05	1.37–6.78	0.006
Age (per year)	1.04	1.00–1.07	0.028
Mammography type (Diagnostic vs. Screening)	1.30	0.69–2.48	0.419

## Data Availability

The data presented in this study are available from the corresponding author upon reasonable request. The data are not publicly available because they contain potentially identifying patient information.
